# Statistics of Rheumatism

**Published:** 1874-06

**Authors:** 

**Affiliations:** St. Thomas’ Hospital


Statistics of Rheumatism.—*By Dr. Peacock, of St. Thomas' Hospital.*—1. Acute forms of rheumatic fever are not common in early life. The chronic forms are almost peculiar to old age. 2. The disease is more common in men than in women. 3. One attack of acute rheumatism predisposes to another. 4. As many as nine attacks were found to have occurred in one patient. 5. In most cases the disease runs a mild course. 6. In none of the reported cases did the temperature rise above 105° Fahr., and in only a few did it reach103° Fahr. The highest temperature was usually found on the day after admission. 7. The greatest risk in the course of the disease arises from cardiac complication. Over thirty-three per cent. of all the cases showed more or less signs of it, and in most instances the heart was found affected at the period of admission. 8. Cardiac complications are most common in early life, and are more frequent in the male than in the female. 9. The cardiac mischief is not directly proportional to the severity of the fever. In a mild case the heart may become affected, while in a severe case it may remain entirely unaffected. 10. Pericarditis is most common in the slight, endocarditis in the severe attacks. 11. Inflammations of the lungs and pleura are not unfrequent in course of acute rheumatism. 12. The treatment of acute cases consisted chiefly of bicarbonate of potash, with or without nitrate. In the sub-acute, iodide of potash and small doses of colchicum were administered. To quiet pain opium was given, and to clean the tongue mercurial purges. Heart complications were combated by blisters and poultices. Convalesence was aided by quinine and iron.

